# Supplementary material for: Computational pathology identifies immune-mediated collagen disruption to predict clinical outcomes in gynecologic malignancies
Source: Commun Med (Lond). 2024 Jan 3;4:2. doi: 10.1038/s43856-023-00428-0 (PMC10764846; doi:10.1038/s43856-023-00428-0)
Supplement: Supplementary file 2 — Supplementary Information [file 43856_2023_428_MOESM2_ESM.pdf]

Supplementary Methods

Patient populations for the study

For genomic analysis, we employed a log_2-transformed fragments per kilobase of mRNA per million reads (FPKM) of the estimated gene expression profiles (level 3, from "IlluminaHiSeq_RNASeqV2" platform) of tumor samples from the TCGA normalized read counts, which aligned to the GRCh38 (V.28) reference genome and their corresponding H&E WSI and clinical data were available.

Overall survival (OS) was defined as the time from diagnosis to death or last follow-up for survivors, while progression-free survival (PFS) was defined as the time from diagnosis to disease progression or death, whichever came first, and was censored for patients without disease progression at the last follow-up. A Cox proportional hazard model[1] (referred to as Cox regression model) with elastic net penalty[2] was trained using CollaTIL features on the D0 cohort to predict OS.

CollaTIL framework

- **Epithelium/Stroma segmentation**

The epithelial and stromal regions were segmented using a pretrained deep learning model with a U-Net architecture, which consisted of 14,788,929 parameters[3]. The model was trained and validated using 212 well-annotated oral cavity tissue microarray images and performed consistently on the three independent test cohorts used in the study[3]. A probability threshold was applied to each pixel to determine its likelihood of being an

epithelial pixel. The resulting probabilistic epithelial mask was converted into a binary mask using a specific probability threshold. Morphological operations, such as removing connected components smaller than 2000 pixels, were applied to the binary mask. Furthermore, dividing sections into epithelium and stromal components assumed that the epithelium component had a high enough proportion of invasive carcinoma, rendering rare benign epithelial components negligible for the model.

- **Nuclei segmentation**

The present study utilized the Hovernet model [4] to automatically segment all nuclei in the image dataset. This model was trained on the Pannuke dataset[5], which includes approximately 200,000 nuclei categorized into five classes to facilitate the task of segmenting and classifying nuclei on H&E WSIs. The Pannuke dataset comprises 19 different tissue types, and the annotations were done in a semi-automated manner by clinical pathologists, ensuring high-quality annotations and minimizing selection bias[5]. The output generated by the Hovernet model is a binary mask that outlines the nuclei regions on the H&E WSI, providing a precise segmentation of the nuclei.

- **TIL identification**

Following the segmentation of each nucleus on the H&E WSI using the Hovernet model, we classified them as either TILs or non-TILs. This classification was achieved using a

pre-trained support vector machine with a linear kernel, which utilized texture, shape, and color-related features derived from the images[6]. Moreover, utilizing the previously identified epithelial and stromal regions, we established four distinct classes of nuclei: epithelial TILs, epithelial non-TILs, stromal TILs, and stromal non-TILs.

- **CollaTIL feature extraction**

Each patient is characterized by a total of 34 features. The Collagen features quantify the disorder in collagen fiber orientations within stromal regions on the H&E WSI. To accomplish this, we partitioned each tile into an array of tumor neighborhoods and used a derivative-of-Gaussian based model to capture fiber orientations by identifying linear structures within the stromal regions. We ensured that the detected linear structures did not overlap with the segmented nuclei by utilizing the nuclei segmentation mask. For each tumor neighborhood, an orientation co-occurrence matrix was constructed based on the set of fiber orientations. Each row and column in this matrix corresponded to an angular bin, ranging from 0 to 17, obtained by discretizing the fiber orientations. Since fiber orientation values fall within the range of 0 to 180 degrees, we chose this discretization as a hyperparameter setting it to 18 bins. The quantitative measurement of Collagen Fiber Orientation Disorder in the Stromal regions was subsequently computed from this matrix using entropy theory, where entropy represents the level of uncertainty in the orientation of collagen fibers within the tumor neighborhood. We extracted first-order statistics (mean, minimum, and maximum) from these feature maps for nine different tumor neighborhood sizes, resulting in 27 features per patient. This methodology is described in more detail in the study by Li et al.[7].

The extracted features from immune architecture characterize the geospatial architecture and interplay of TIL and non-TIL nuclei on the H&E WSI. We categorized each tile into three compartments based on the ratio of epithelial to stromal tissue: epithelium (ratio of epithelium to stroma > 3), stroma (ratio of stroma to epithelium > 3), and invasive tumor front (ratio of epithelium to stroma between ⅓ and 3). For each tile within the individual tissue compartments, we extracted features related to the geospatial architecture and interplay of TIL and non-TIL nuclei. This was done by classifying each segmented nuclei as either TIL or non-TIL using a pre-existing machine learning model[6]. Based on the previously detected epithelial and stromal regions, four different classes of nuclei were defined: epithelial TILs, epithelial non-TILs, stromal TILs, and stromal non-TILs. We characterized the spatial arrangement and density of TILs by constructing clusters of each nuclei class based on proximity. A nucleus is linked to another nucleus of the same type if the distance between them is below a threshold (defined as 20 microns in the study by Azarianpour et al.[8]). The final feature vector was obtained by computing six metrics (mean, median, minimum, maximum, range, and variance) for each feature across the total number of epithelial, stromal, and invasive tumor front tiles, resulting in 21,096 features per patient. In the study conducted by Azarianpour et al.[8], the authors trained a survival model using 51 patients from the ovarian cancer cohort obtained from TCGA using these features and identified 7 features associated with survival outcomes. In the present study, we selected the same 7 features for analysis, considering their demonstrated prognostic value.

Steps involved in extracting features from collagen component

The steps involved in extracting features from collagen component are as follows:

- Detecting collagen fibers
- Find the orientation of the detected collagen fibers
- Discretize the orientations in 18 bins (0 to 17)
- Then tumor neighborhoods of nine different sizes (200x200-pixel, 250x250-pixel, 300x300-pixel, 350x350-pixel, 400x400-pixel, 450x450-pixel, 500x500-pixel, 550x550-pixel, 600x600-pixel) move across the extracted tiles of whole slide images in a sliding window way, calculate the disorder of collagen fiber orientations by constructing an orientation co-occurrence matrix within each tumor neighborhood
- Quantitative measurement of the disorder in collagen fiber orientations was measured using the orientation co-occurrence matrix using entropy theory

Step 1: Detecting collagen fibers using derivative of Gaussian model

- We used the derivative of Gaussian based model in Matlab to detect the linear structures in the image, which are the prevalent morphology phenotype exhibited by collagen fibers on H&E images.

Step 2: Orientation of detected collagen fibers (regionprops function in Matlab)

- We use the collagen fibers that are detected by the derivative-of-Gaussian model. Essentially for each pixel we compute the x-gradient, y-gradient and that gives us the orientation (Orientation (θ) = atan2(y-gradient, x-gradient))
- For each collagen fiber, we create the histogram of orientations for each pixel as computed above and find the orientation that occurs more frequently that is considered as the orientation for that collagen fiber.

Step 3: Discretize the orientations in 18 bins from 0 to 17

Step 4: Orientation co-occurrence matrix

Divide each tile extracted from the whole slide image in tumor neighborhoods of nine different sizes (200x200-pixel, 250x250-pixel and so on). Within each neighborhood, compute the orientation co-occurrence matrix where each row and column of the matrix denote the frequency of orientations having value row or column.

Step 5: Quantitative measurement of the disorder in collagen fiber orientations

After obtaining the orientation co-occurrence matrix for each neighborhood, we essentially use this matrix to compute the disorder in collagen fiber orientations using entropy theory.

Univariate analysis, multivariable analysis, and integrated nomogram

After performing univariate and multivariable analysis of clinical and pathological variables on validation cohorts (D1-D8) for predicting OS/PFS, we determined the clinical variables that should be incorporated into the construction of a clinical histomorphometric nomogram along with CollaTIL. Results of the multivariable analysis indicated that CollaTIL risk score (obtained during univariate analysis of CollaTIL) and stage were the two variables that should be used for training the Cox regression model on the D0 cohort. We tested the nomogram in the validation cohorts (D1-D8), and found that it was associated with OS in all chemotherapy and radiotherapy-treated cohorts and with PFS in the chemotherapy-treated cohort (D1, p=0.04, HR=2.2, 95% CI=1.05-4.61; D2, p=0.003, HR=3.11, 95% CI=1.62-5.99; D3, p=0.04, HR=5.76, 95% CI=2.02-16.5; D8, p=0.043, HR=3.07, 95% CI=1.01-9.85). Moreover, in the immunotherapy-treated validation cohorts, it was associated with PFS (D5, D6, and D7, p=0.018, HR=2.72, 95% CI=1.36-5.42).

Prognostic value of signatures (Collagen component, Immune component, Collagen+Immune components) in the chemotherapy, radiotherapy, and immunotherapy-treated validation cohorts

The signature (Collagen features alone) was not associated with OS in chemotherapy and radiotherapy-treated patients and PFS in chemotherapy-treated patients (chemotherapy CSCC (D1, p=0.63, HR=1.2, 95% CI=0.57-2.52, c-index=0.59), radiotherapy CSCC (D2, p=0.1, HR=1.74, 95% CI=0.91-3.35, c-index=0.59), chemotherapy EC (D3, p=0.38, HR=0.676, 95% CI=0.63-1.71, c-index=0.41), chemotherapy HGSOC (D8, p=0.54, HR=1.59, 95% CI=0.422-5.96, c-index=0.71)). In the cohort treated with no therapy (D4), the signature was associated with OS (p=0.04, HR=3.1, 95% CI=1.02-15.6, c-index=0.82). In the immunotherapy-treated cohorts (D5, D6 and D7), the signature was not associated with PFS (p=0.58, HR=0.823, 95% CI=0.42-1.62, c-index=0.51).

The signature (Immune features alone) was associated with OS in chemotherapy and radiotherapy-treated patients but not with PFS in chemotherapy-treated patients (chemotherapy CSCC (D1, p=0.001, HR=4.3, 95% CI=2.05-9.03, c-index=0.7), radiotherapy CSCC (D2, p=0.007, HR=2.96, 95% CI=1.52-5.73, c-index=0.69), chemotherapy EC (D3, p=0.037, HR=3.38, 95% CI=1.34-8.51, c-index=0.66), chemotherapy HGSOC (D8, p=0.08, HR=3.07, 95% CI=0.9-9.85, c-index=0.57)). In the cohort treated with no therapy (D4), the signature was not associated with OS (p=0.08,

HR=3.11, 95% CI=0.727-13.3, c-index=0.75). In the immunotherapy-treated cohorts (D5, D6 and D7), the signature was also not associated with PFS (p=0.122, HR=1.79, 95% CI=0.9-3.54, c-index=0.58).

The CollaTIL signature (both Collagen and Immune features) was associated with OS or PFS in various validation cohorts. Specifically, in the chemotherapy-treated validation cohorts (D1, D3 and D8), it was associated with OS in D1 (p=0.0256, HR=2.54, 95% CI=1.21-5.34, c-index=0.72), D3 (p=0.0213, HR=7.36, 95% CI=2.73-19.8, c-index=0.7), and PFS in D8 (p=0.0434, HR=3.07, 95% CI=1.01-9.85, c-index=0.74). In the radiotherapy-treated validation cohort (D2), it was also associated with OS (p=0.006, HR=2.87, 95% CI=1.49-5.53, c-index=0.72). Moreover, the signature identified high risk patients who had significantly worse PFS than low-risk patients in the immunotherapy-treated validation cohorts (D5, D6, and D7, p=0.0184, HR=2.72, 95% CI=1.36-5.42, c-index=0.65).

Comparison of TIL detection models

We conducted an experiment to determine which TIL classification model we should use for the present work: SVM[6] or Hover-Net[4]. For this, we randomly selected 80 tiles of size 3000x3000-pixel from the training cohort (D0). We applied both approaches to the tiles and asked two pathologists to select the model that provided the best TIL detection. One of the pathologists stated that the SVM model performed better in 52% of the tiles, Hover-Net in 28%, and there was not a significant difference in the remaining 20%. The second pathologist noted that the SVM was better in 75% of the cases, Hover-Net in 19%, and no significant difference was observed in 6% of the cases. We asked the pathologists for their overall impression of the models, and both of them agreed that while Hover-Net was very precise, it had lower recall. For this reason, we chose to use the SVM model.

Signature Index

In Single Sample Gene Set Enrichment Analysis (ssGSEA) enrichment analysis tools, the term "signature index" typically refers to a numerical score or metric that quantifies the extent to which a particular gene expression signature or gene set is enriched or depleted in a single sample or specimen. It summarizes certain characteristics or properties of data. In ssGSEA, the "signature index" assigned to a sample, indicating how closely its gene expression profile aligns with the gene expression pattern of a

particular gene set or signature. It quantifies the degree of enrichment (positive index) or depletion (negative index) of the genes within the signature in the sample[9].

We used signature index values to gain information about the pathways that are activated or suppressed in individual samples, helping us understand the underlying biology of the high risk and low risk cohorts in the context of the identified gene sets.

False discovery rate (FDR)

FDR stands for False Discovery Rate. In multiple hypothesis testing of gene expression data, the FDR addresses a specific challenge that traditional p-values or family-wise error rates (FWER) do not fully account for the control of false positives when testing multiple hypotheses simultaneously[10].

Supplementary Tables

Supplementary Table 1. Summary of clinical and pathological features for the patients in the whole dataset

Cohort (N)	Therapy	Cancer	#slides per patient	Histology (FIGO Stage I, II, III, IV, missing)	#Censored	Mean age ± std (years)	Site	Clinical endpoint of interest
D0 (95), Training	Chemotherapy	HGSOC	1	High-grade serous ovarian carcinoma (Stage I: 2, Stage II: 4, Stage III: 68, Stage IV: 20, Missing: 1)	32 (33.7%)	60.28 ± 14.23	TCGA	OS
D1 (134), Validation	Chemotherapy	CSCC	1	Squamous cell carcinoma (Stage I: 72, Stage II: 30, Stage III: 22, Stage IV: 8, Missing: 2)	106 (79%)	48.39 ± 13.35	TCGA	OS
D2 (128), Validation	Radiotherapy	CSCC	1	Squamous cell carcinoma (Stage I: 65, Stage II: 30, Stage III: 19, Stage IV: 11, Missing: 3)	92 (71.9%)	48.27 ± 13.39	TCGA	OS
D3 (32), Validation	Chemotherapy	EC	1-4	Endometroid, Serous, Mixed, Clear cell (Stage I: 14, Stage II: 2,	13 (40.6%)	62.19 ± 4.5	UH	OS

				Stage III: 10,<br>Stage IV: 6,<br>Missing: 0)				
D4 (26),<br>Validation	No therapy	EC	1-4	Endometroid, Clear cell<br>(Stage I: 26,<br>Stage II: 0,<br>Stage III: 0,<br>Stage IV: 0,<br>Missing: 0)	18 (69.2%)	61.15<br>± 4.9	UH	OS
D5 (14),<br>Validation	Immunotherapy<br>(recurrent setting)	HGSOC	1	High-grade serous ovarian cancer<br>(Stage I: 1,<br>Stage II: 2,<br>Stage III: 5,<br>Stage IV: 5,<br>Missing: 1)	3 (21.43%)	63.3<br>± 13.1	CCF	PFS
D6 (7),<br>Validation	Immunotherapy<br>(recurrent setting)	CSCC	1	Squamous cell carcinoma<br>(Stage I: 0,<br>Stage II: 0,<br>Stage III: 4,<br>Stage IV: 3,<br>Missing: 0)	3 (42.86%)	63.1<br>± 13.7	CCF	PFS
D7 (27),<br>Validation	Immunotherapy<br>(recurrent setting)	EC	1	Endometrioid,<br>Serous, Clear cell<br>(Stage I: 12,<br>Stage II: 0,<br>Stage III: 8,<br>Stage IV: 5,<br>Missing: 2)	12 (44.4%)	71.3<br>± 9.1	CCF	PFS
D8 (30),<br>Validation	Chemotherapy	HGSOC	1	High-grade serous ovarian cancer<br>(Stage I: 0,<br>Stage II: 0,<br>Stage III: 17,	18 (60%)	60.73<br>± 8.64	MSKCC	PFS

				Stage IV: 13, Missing: 0)				

*TCGA, The Cancer Genome Atlas; UH, University Hospitals; CCF, Cleveland Clinic; MSKCC, Memorial Sloan Kettering Cancer Center; EC, endometrial carcinoma; HGSOC, high grade serous ovarian carcinoma; CSCC, cervical squamous cell carcinoma; FIGO, International Federation of Gynecology and Obstetrics; OS, overall survival; PFS, progression-free survival.

*For D3 and D4 cohorts having multiple tissue slides per patient, the most representative tissue slide selected by the pathologist (Dr. Stefanie Avril) was used for analysis.

Supplementary Table 2. Features from TIL component

**Feature index**	**Feature description**
1	Ratio of non-TILs density to the surrounding (20 microns proximity) TILs in the epithelium compartment
2	Number of epithelial TIL clusters surrounding (20 microns proximity) a non-TIL cluster in the epithelium compartment
3	Presence percentage (ratio of present clusters to total number of clusters) of stromal non-TIL clusters being around another non-TIL cluster in the stromal compartment
4	Intersected area of clusters of epithelial TILs and non-TILs in invasive tumor front compartment
5	Minimum area of stromal TIL clusters in invasive tumor front compartment
6	Range of area of epithelial non-TIL clusters in invasive tumor front compartment
7	Range of density of TIL clusters to the surrounding non-TIL ones in stroma

Supplementary Table 3. Features from collagen component

Feature index	Feature description
1	Mean entropy value of the collagen fiber orientation disorder feature map using 200x200-pixel neighborhood
2	Minimum entropy value of the collagen fiber orientation disorder feature map using 200x200-pixel neighborhood
3	Maximum entropy value of the collagen fiber orientation disorder feature map using 200x200-pixel neighborhood
4	Mean entropy value of the collagen fiber orientation disorder feature map using 250x250-pixel neighborhood
5	Minimum entropy value of the collagen fiber orientation disorder feature map using 250x250-pixel neighborhood
6	Maximum entropy value of the collagen fiber orientation disorder feature map using 250x250-pixel neighborhood
7	Mean entropy value of the collagen fiber orientation disorder feature map using 300x300-pixel neighborhood
8	Minimum entropy value of the collagen fiber orientation disorder feature map using 300x300-pixel neighborhood
9	Maximum entropy value of the collagen fiber orientation disorder feature map using 300x300-pixel neighborhood
10	Mean entropy value of the collagen fiber orientation disorder feature map using 350x350-pixel neighborhood
11	Minimum entropy value of the collagen fiber orientation disorder feature map using 350x350-pixel neighborhood
12	Maximum entropy value of the collagen fiber orientation disorder feature map using 350x350-pixel neighborhood
13	Mean entropy value of the collagen fiber orientation disorder feature map using 400x400-pixel neighborhood

14	Minimum entropy value of the collagen fiber orientation disorder feature map using 400x400-pixel neighborhood
15	Maximum entropy value of the collagen fiber orientation disorder feature map using 400x400-pixel neighborhood
16	Mean entropy value of the collagen fiber orientation disorder feature map using 450x450-pixel neighborhood
17	Minimum entropy value of the collagen fiber orientation disorder feature map using 450x450-pixel neighborhood
18	Maximum entropy value of the collagen fiber orientation disorder feature map using 450x450-pixel neighborhood
19	Mean entropy value of the collagen fiber orientation disorder feature map using 500x500-pixel neighborhood
20	Minimum entropy value of the collagen fiber orientation disorder feature map using 500x500-pixel neighborhood
21	Maximum entropy value of the collagen fiber orientation disorder feature map using 500x500-pixel neighborhood
22	Mean entropy value of the collagen fiber orientation disorder feature map using 550x550-pixel neighborhood
23	Minimum entropy value of the collagen fiber orientation disorder feature map using 550x550-pixel neighborhood
24	Maximum entropy value of the collagen fiber orientation disorder feature map using 550x550-pixel neighborhood
25	Mean entropy value of the collagen fiber orientation disorder feature map using 600x600-pixel neighborhood
26	Minimum entropy value of the collagen fiber orientation disorder feature map using 600x600-pixel neighborhood
27	Maximum entropy value of the collagen fiber orientation disorder feature map using 600x600-pixel neighborhood

Supplementary Table 4. Quality check results of Epithelium/Stroma segmentation, Nuclei segmentation, Collagen fiber segmentation, and TIL detection methods

**Task**	**Pathologist 1, Dr. Stefanie Avril**<br><br>**(% of tiles belonging to good/fair category)**	**Pathologist 2, Dr. Mojgan Mokhtari**<br><br>**(% of tiles belonging to good/fair category)**
Epithelium/Stroma segmentation	90%	94%
Nuclei segmentation	100%	100%
Collagen fiber segmentation	90%	92%
TIL detection	90%	94%

Supplementary Table 5. Hazard Ratios and P-values from univariate and multivariable analysis of overall survival (D1-D4) and progression-free survival (D5-D8)

Clinicopathological Variables	**D1 HR (p, 95% CI)**	**D2 HR (p, 95% CI)**	**D3 HR (p, 95% CI)**	**D4 HR (p, 95% CI)**	**D5, D6, D7 HR (p, 95% CI)**	**D8 HR (p, 95% CI)**
**Univariate Analysis**						
Age (>60 years vs. <=60 years)	**HR = 3.21 (p=0.001, CI=1.19-8.63)**	HR=1.38 (p=0.401, CI=0.604-3.14)	**HR=3.57 (p=0.0295, CI=1.42-8.93)**	HR=1.38 (p=0.681, CI=0.311-6.13)	HR=0.879 (p=0.735, CI=0.401-1.92)	HR=1.7 (p=0.355, CI=0.547-5.3)
FIGO stage (stage III/IV vs. stage I/II)	**HR=2.66 (p=0.0085, CI=1.04-6.75)**	**HR=2.24 (p=0.019, CI=1.01-5.21)**	HR=1.59 (p=0.295, CI=0.646-3.92)	HR=1 (p=1, CI=0-Inf)	HR=1.66 (p=0.153, CI=0.848-3.23)	HR=1 (p=1, CI=0-Inf)
Tumor grade (high vs. low)	*no data	*no data	HR=2.26 (p=0.182, CI=0.85-6.04)	HR=3.13 (p=0.08, CI=0.62-18)	HR=0.985 (p=0.972, CI=0.5-2.26)	*no data
CollaTIL (high vs. low)	**HR=2.54 (p=0.0256, CI=1.21-5.34)**	**HR=2.87 (p=0.006, CI=1.49-5.53)**	**HR=7.36 (p=0.0213, CI=2.73-19.8)**	**HR=4.39 (p=0.0259, CI=1.07-18)**	**HR=2.72 (p=0.0184, CI=1.36-5.42)**	**HR=3.07 (p=0.0434, CI=1.01-9.85)**
Molecular subtypes (Cnhigh vs. Cnlow)	**-**	**-**	HR=1.58 (p=0.3, CI=0.64-3.89)	HR=1.06 (p=0.9, CI=0.2-5.4)	**-**	**-**

HPV dependency data (HPV+ vs. HPV-)	HR=1.18 (p=0.38, CI=1.1-6.9)	HR=1.47 (p=0.7, CI=1.1-7.73)	-	-	**-**	**-**
**Multivariable Analysis**						
Clinicopathological Variables	**D1 HR (p, 95% CI)**	**D2 HR (p, 95% CI)**	**D3 HR (p, 95% CI)**	**D4 HR (p, 95% CI)**	**D5, D6, D7 HR (p, 95% CI)**	**D8 HR (p, 95% CI)**
Age (>60 years vs. <=60 years)	**HR=2.62 (p=0.02, CI=1.18-5.8)**	HR=1.28 (p=0.53, CI=0.59-2.8)	HR=3.39 (p=0.06, CI=0.95-12)	HR=1.08 (p=1, CI=0-Inf)	HR=1.09 (p=0.83, CI=0.5-2.4)	HR=1.43 (p=0.58, CI=0.4-5.2)
FIGO stage (FIGO IV vs. FIGO III vs. FIGO II vs. FIGO I)	**HR=1.46 (p=0.04, CI=1.03-2.1)**	**HR=1.58 (p=0.01, CI=1.15-2.2)**	**HR=1.56 (p=0.048, CI=1.01-2.5)**	HR=1 (p=1, CI=0-Inf)	HR=1.21 (p=0.2, CI=0.9-1.6)	HR=1.09 (p=0.9, CI=0.3-4)
CollaTIL (high vs. low)	**HR=2.58 (p=0.03, CI=1.09-6.1)**	**HR=3.25 (p=0.0001, CI=1.45-7.3)**	HR=6.16 (p=0.08, CI=0.79-48)	HR=9.35 (p=1, CI=0-Inf)	**HR=2.74 (p=0.03, CI=1.13-6.7)**	HR=1 (p=1, CI=0-Inf)

*HR, Hazard Ratio; CI, Confidence Interval

*Bolded values indicate significant Hazard Ratios and P-values.

*FIGO, International Federation of Gynecology and Obstetrics.

Supplementary Figures

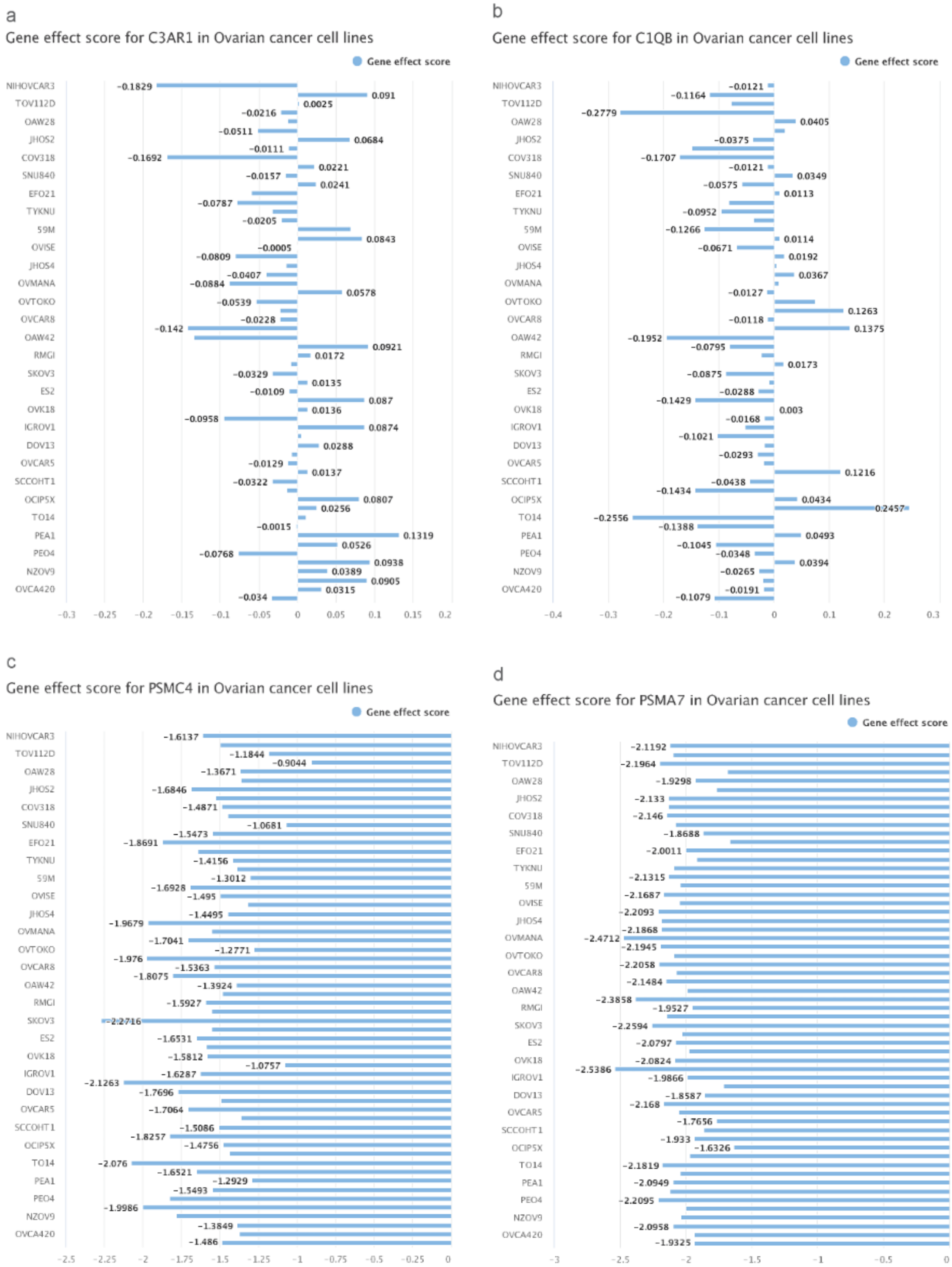

Supplementary Figure 1: Knockout of Macrophages, and Amino acid-associated genes indicating cellular growth inhibition or cell death. a-b Knockout of amino Macrophage genes of *C3AR1, and C1QB* indicating cellular growth inhibition or cell death as the negative scores imply cell growth inhibition and/or death following gene knockout. **c-d** Knockout of amino acid-associated genes of *PSMC4 and PSMA7* indicating cellular growth inhibition or cell death as the negative scores imply cell growth inhibition and/or death following gene knockout. Scores are normalized such that nonessential genes have a median score of 0 and independently identified common essentials have a median score of -1. Gene Effect scores were influenced by Chronos[11]

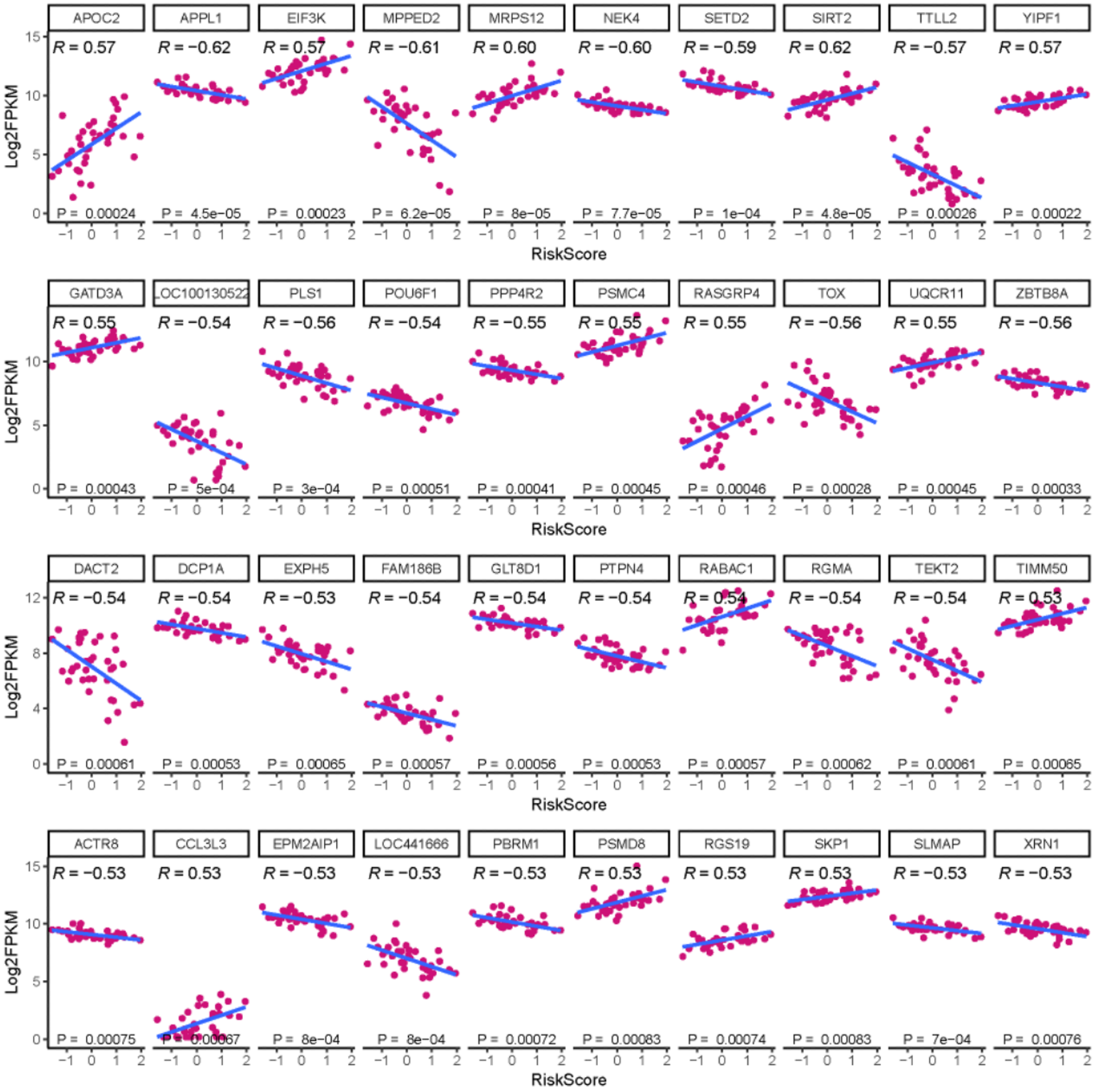

Supplementary Figure 2: Expression of the genes that are strongly correlated with the risk scores predicted based on CollaTIL. Y axis indicates $\log_2$ FPKM values of the mRNA expression of the top correlated genes on TCGA dataset. X axis indicates the risk-scores predicted based on CollaTIL. R indicates Pearson correlation coefficients.

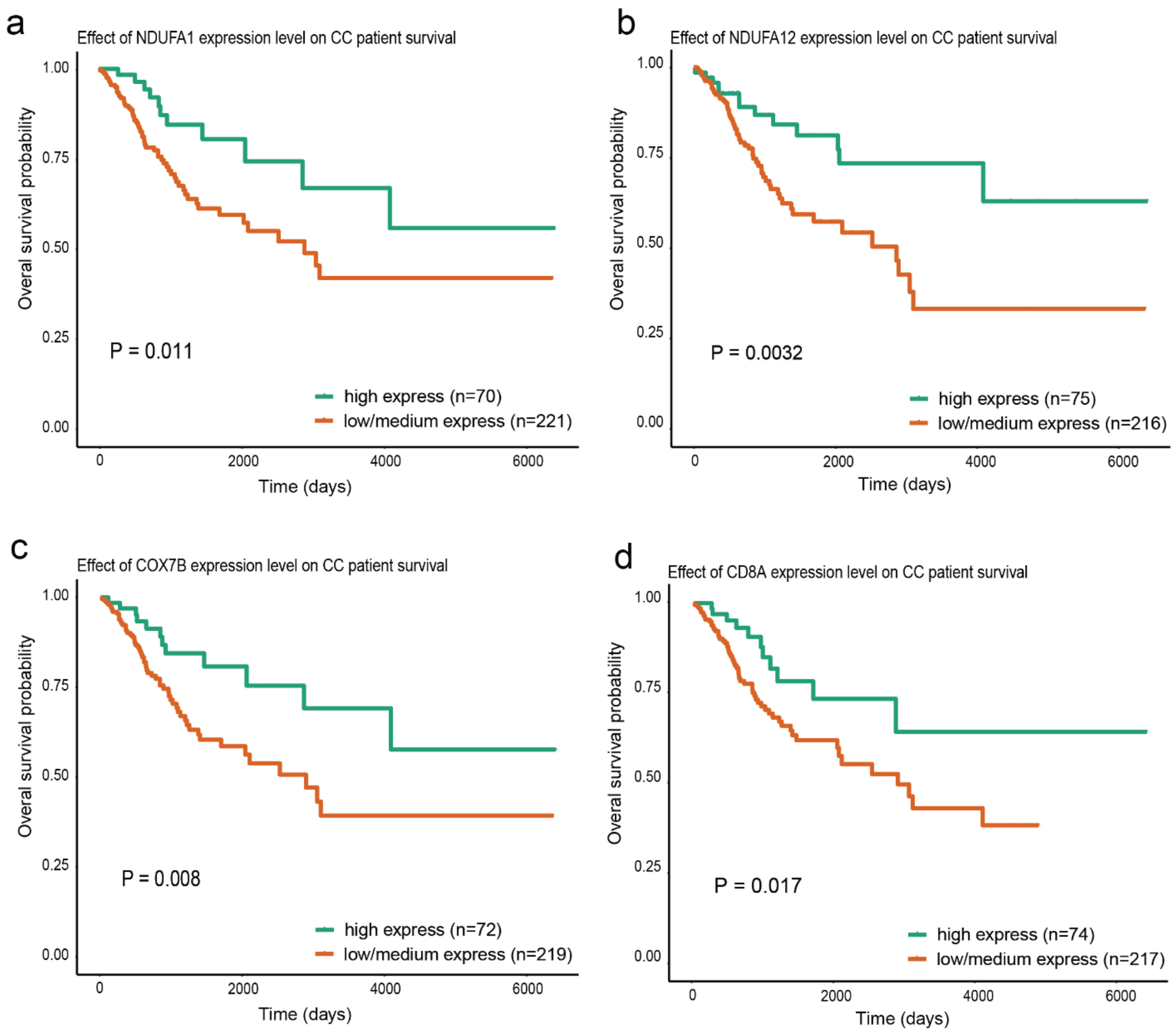

Supplementary Figure 3: Kaplan-Meier curves of TCA-Cycle related genes, *NDUFA1, NDUFA12, COX7B* and *CD8A* that are significantly upregulated and associated with a better prognosis in CSCC patients. a NDUFA1**. b** NDUFA12**. c** COX7B**. d** CD8A**.** The statistical significance of survival rates differences between high expressed and low expressed categories was determined using the LogRank test (P). HGSOC, Ovarian high grade serous carcinoma; CSCC, Cervical squamous cell carcinoma; OS, overall survival; PFS, progression-free survival.

Supplementary References

1. Cox, D. R. Regression Models and Life-Tables. *Journal of the Royal Statistical Society: Series B (Methodological)* **34**, 187–202 (1972).
2. Wu, Y. Elastic net for Cox's proportional hazards model with a solution path algorithm. *Stat Sin* **22**, 271–294 (2012).
3. Wu, Y. *et al.* A machine learning model for separating epithelial and stromal regions in oral cavity squamous cell carcinomas using H&E-stained histology images: A multi-center, retrospective study. *Oral Oncol* **131**, (2022).
4. Graham, S. *et al.* Hover-Net: Simultaneous segmentation and classification of nuclei in multi-tissue histology images. *Med Image Anal* **58**, (2019).
5. Gamper, J. *et al.* PanNuke Dataset Extension, Insights and Baselines. (2020).
6. Romero Castro, E. *et al.* A watershed and feature-based approach for automated detection of lymphocytes on lung cancer images. in 26 (SPIE-Intl Soc Optical Eng, 2018). doi:10.1117/12.2293147.
7. Li, H. *et al.* Collagen fiber orientation disorder from H&E images is prognostic for early stage breast cancer: clinical trial validation. *NPJ Breast Cancer* **7**, (2021).
8. Azarianpour, S. *et al.* Computational image features of immune architecture is associated with clinical benefit and survival in gynecological cancers across treatment modalities. *J Immunother Cancer* **10**, (2022).
9. Barbie, D. A. *et al.* Systematic RNA interference reveals that oncogenic KRAS-driven cancers require TBK1. *Nature* **462**, 108–112 (2009).
10. Benjamini, Y. & Hochberg, Y. *Controlling the False Discovery Rate: A Practical and Powerful Approach to Multiple Testing*. *Source: Journal of the Royal Statistical Society. Series B (Methodological)* vol. 57 (1995).
11. Dempster, J. M. *et al.* Chronos: a cell population dynamics model of CRISPR experiments that improves inference of gene fitness effects. *Genome Biol* **22**, (2021).
