# Supplementary material for: Computational pathology identifies immune-mediated collagen disruption to predict clinical outcomes in gynecologic malignancies
Source: Commun Med (Lond). 2024 Jan 3;4:2. doi: 10.1038/s43856-023-00428-0 (PMC10764846; doi:10.1038/s43856-023-00428-0)
Supplement: Supplementary file 3 — Description of Additional Supplementary Files [file 43856_2023_428_MOESM3_ESM.pdf]

Description of Additional Supplementary Files

File Name: Supplementary Data 1

Description: List of gene sets for Macrophage gene signature and Amino acid signature.
