# Supplementary material for: Computational pathology identifies immune-mediated collagen disruption to predict clinical outcomes in gynecologic malignancies
Source: Commun Med (Lond). 2024 Jan 3;4:2. doi: 10.1038/s43856-023-00428-0 (PMC10764846; doi:10.1038/s43856-023-00428-0)
Supplement: Supplementary file 5 — Reporting Summary [file 43856_2023_428_MOESM5_ESM.pdf]

nature portfolio

Corresponding author(s): Anant Madabhushi

Last updated by author(s): Nov 19, 2023

Reporting Summary

Nature Portfolio wishes to improve the reproducibility of the work that we publish. This form provides structure for consistency and transparency in reporting. For further information on Nature Portfolio policies, see our Editorial Policies and the Editorial Policy Checklist.

Statistics

For all statistical analyses, confirm that the following items are present in the figure legend, table legend, main text, or Methods section.

n/a	Confirmed
☐	☒ The exact sample size (*n*) for each experimental group/condition, given as a discrete number and unit of measurement
☐	☒ A statement on whether measurements were taken from distinct samples or whether the same sample was measured repeatedly
☐	☒ The statistical test(s) used AND whether they are one- or two-sided<br>*Only common tests should be described solely by name; describe more complex techniques in the Methods section.*
☐	☒ A description of all covariates tested
☐	☒ A description of any assumptions or corrections, such as tests of normality and adjustment for multiple comparisons
☐	☒ A full description of the statistical parameters including central tendency (e.g. means) or other basic estimates (e.g. regression coefficient) AND variation (e.g. standard deviation) or associated estimates of uncertainty (e.g. confidence intervals)
☐	☒ For null hypothesis testing, the test statistic (e.g. F, t, r) with confidence intervals, effect sizes, degrees of freedom and P value noted<br>*Give P values as exact values whenever suitable.*
☐	☒ For Bayesian analysis, information on the choice of priors and Markov chain Monte Carlo settings
☐	☒ For hierarchical and complex designs, identification of the appropriate level for tests and full reporting of outcomes
☐	☒ Estimates of effect sizes (e.g. Cohen's *d*, Pearson's *r*), indicating how they were calculated

Our web collection on statistics for biologists contains articles on many of the points above.

Software and code

Policy information about availability of computer code

Data collection	MATLAB, Python, and R were used to perform the data collection and analysis. The codes were made available in Github. Python 3.11 was used. MATLAB 2021b was the main version. R version 4.0. Different packages from python were used that can be seen in the scripts located at Github (https://github.com/arp95/collatil_biomarker_gyn/)
Data analysis	MATLAB, Python, and R were used to perform the data collection and analysis. The codes were made available in Github. Python 3.11 was used. MATLAB 2021b was the main version. R version 4.0. Different packages from python were used that can be seen in the scripts located at Github (https://github.com/arp95/collatil_biomarker_gyn/)

For manuscripts utilizing custom algorithms or software that are central to the research but not yet described in published literature, software must be made available to editors and reviewers. We strongly encourage code deposition in a community repository (e.g. GitHub). See the Nature Portfolio guidelines for submitting code & software for further information.

Data

Policy information about availability of data

All manuscripts must include a data availability statement. This statement should provide the following information, where applicable:

- Accession codes, unique identifiers, or web links for publicly available datasets
- A description of any restrictions on data availability
- For clinical datasets or third party data, please ensure that the statement adheres to our policy

D0, D1, D2 were generated by TCGA Research Network (http://cancergenome.nih.gov/) and they have made them publicly available. D8 was generated by Memorial Sloan Kettering Cancer Center and is made publicly available at Synapse (Sage Bionetworks) under accession code syn25946117. Since the cases from the involved institutions are protected through institutional compliance, the clinical repository of cases can only be shared per specific institutional review board (IRB) requirements. Upon reasonable request, a data sharing agreement can be initiated between the interested parties and the clinical institution following institution-specific guidelines. This applies to the cohort known as D3, D4, D5, D6, and D7.

Code Availability: Code can be found on GitHub (https://github.com/arp95/collatil_biomarker_gyn/)

Research involving human participants, their data, or biological material

Policy information about studies with human participants or human data. See also policy information about sex, gender (identity/presentation), and sexual orientation and race, ethnicity and racism.

Reporting on sex and gender	n/a
Reporting on race, ethnicity, or other socially relevant groupings	n/a
Population characteristics	n/a
Recruitment	n/a
Ethics oversight	n/a

Note that full information on the approval of the study protocol must also be provided in the manuscript.

Field-specific reporting

Please select the one below that is the best fit for your research. If you are not sure, read the appropriate sections before making your selection.

☒ Life sciences ☐ Behavioural & social sciences ☐ Ecological, evolutionary & environmental sciences

For a reference copy of the document with all sections, see nature.com/documents/nr-reporting-summary-flat.pdf

Life sciences study design

All studies must disclose on these points even when the disclosure is negative.

Sample size	Initial sample size of 493. Sample size was determined as available collection of collaboration with different institutions and partners.
Data exclusions	Data exclusion was primary due to clinical information available to the histology images used and the quality of the images.
Replication	The approach can be reproduced using the available data that is publicly online. The scripts and codes can be used and run to reproduce the work.
Randomization	There was not randomization.
Blinding	There was not blinding.

Reporting for specific materials, systems and methods

We require information from authors about some types of materials, experimental systems and methods used in many studies. Here, indicate whether each material, system or method listed is relevant to your study. If you are not sure if a list item applies to your research, read the appropriate section before selecting a response.

Materials & experimental systems

n/a	Involved in the study
☒	☐ Antibodies
☒	☐ Eukaryotic cell lines
☒	☐ Palaeontology and archaeology
☒	☐ Animals and other organisms
☐	☒ Clinical data
☒	☐ Dual use research of concern
☒	☐ Plants

Methods

n/a	Involved in the study
☒	☐ ChIP-seq
☒	☐ Flow cytometry
☒	☐ MRI-based neuroimaging

Clinical data

Policy information about clinical studies

All manuscripts should comply with the ICMJE guidelines for publication of clinical research and a completed CONSORT checklist must be included with all submissions.

Clinical trial registration: n/a

Study protocol: n/a

Data collection: We analyzed 493 patients diagnosed with High Grade Serous Carcinoma (HGSOC, n=139), Cervical Squamous Cell Carcinoma (CSCC, n=269), or Endometrial Carcinoma (EC, n=85) from various sites, including The Cancer Genome Atlas (TCGA, n=357), University Hospitals (UH, n=58), Cleveland Clinic (CCF, n=48), and Memorial Sloan Kettering Cancer Center (MSKCC, n=30). For the patients from TCGA diagnosed with HGSOC (D0, n=95), tissue samples were obtained through cytoreductive surgery before the initiation of chemotherapy. Similarly, for the patients from TCGA diagnosed with CSCC (D1 and D2, n=262), tissue samples were obtained through cytoreductive surgery before the initiation of chemotherapy or radiotherapy. All patients from UH (D3 and D4, n=58) underwent surgery (hysterectomy with bilateral salpingo-oophorectomy), the standard treatment for EC. The patients in these cohorts who were considered as intermediate- or high-risk for recurrence received chemotherapy following surgery (D3, n=32). The CCF cohorts (D5, D6, and D7) included 48 patients treated with immunotherapy agents, including pembrolizumab, nivolumab±ipilimumab, and avelumab, all in the recurrent setting (D5 for HGSOC, D6 for CSCC, and D7 for EC). All patients from MSKCC (D8, n=30) were diagnosed with HGSOC and underwent primary debulking surgery.

Outcomes: Outcomes related to overall survival (OS) or progression-free survival (PFS).

Plants

Seed stocks: n/a

Novel plant genotypes: n/a

Authentication: n/a
